# Exact solutions for the Cahn–Hilliard equation in terms of Weierstrass-elliptic and Jacobi-elliptic functions

**DOI:** 10.1038/s41598-024-62961-9

**Published:** 2024-06-07

**Authors:** Akhtar Hussain, Tarek F. Ibrahim, F. M. Osman Birkea, Abeer M. Alotaibi, Bushra R. Al-Sinan, Herbert Mukalazi

**Affiliations:** 1 Abdus Salam School of Mathematical Sciences, Government College University, 68-B New Muslim Town, Lahore, 54600 Pakistan; 2 Department of Mathematics, Faculty of Sciences and Arts (Mahayel), King Khalid University, Abha, Saudi Arabia; 3 Department of Mathematics, Faculty of Science, Northern Border University, Arar, Saudi Arabia; 4 Department of Mathematics, Faculty of Science, University of Tabuk, P. O. Box 741, 71491 Tabuk, Saudi Arabia; 5 Department of Administrative and Financial Sciences, Nairiyah College, University of Hafr Al-Batin, 31991 Hafr Al-Batin, Saudi Arabia; 6 Department of Mathematics and Statistics, Kyambogo University, Kampala, Uganda

**Keywords:** Convective–diffusive Cahn–Hilliard equation, Soliton solutions, The F-expansion method, Jacobi-elliptic functions, Mathematics and computing, Optics and photonics, Physics

## Abstract

Despite the historical position of the F-expansion method as a method for acquiring exact solutions to nonlinear partial differential equations (PDEs), this study highlights its superiority over alternative auxiliary equation methods. The efficacy of this method is demonstrated through its application to solve the convective–diffusive Cahn–Hilliard (cdCH) equation, describing the dynamic of the separation phase for ternary iron alloys (Fe–Cr–Mo) and (Fe–X–Cu). Significantly, this research introduces an extensive collection of exact solutions by the auxiliary equation, comprising fifty-two distinct types. Six of these are associated with Weierstrass-elliptic function solutions, while the remaining solutions are expressed in Jacobi-elliptic functions. I think it is important to emphasize that, exercising caution regarding the statement of the term ’new,’ the solutions presented in this context are not entirely unprecedented. The paper examines numerous examples to substantiate this perspective. Furthermore, the study broadens its scope to include soliton-like and trigonometric-function solutions as special cases. This underscores that the antecedently obtained outcomes through the recently specific cases encompassed within the more comprehensive scope of the present findings.

## Introduction

Nonlinear equations constitute a pivotal tool in the modeling of intricate phenomena within the domain of nonlinear sciences. In recent decades, the scientific community, comprising physicists and mathematicians, has elucidated the proficiency of nonlinear differential equations in representing a myriad of nonlinear occurrences across a spectrum of applied sciences. These encompass optics, optical fibers, birefringent fibers, plasma physics, elastic media, geology, human biology, fluid dynamics, ecology, engineering, fluid mechanics, applied mathematics, computer science, medicine, and diverse other disciplines^1–9^. The Cahn–Hilliard (CH) model^10–14^ assumes a fundamental role in elucidating phase separation phenomena within physical systems, notably in the context of alloys. Noteworthy is the recent resurgence of interest in the CH equation, as it demonstrates applicability in modeling the dynamics of fluid separation under specific conditions. This resurgence underscores the universal applicability and scientific significance of nonlinear equations in capturing intricate phenomena across diverse scientific domains.

This investigation focuses on scrutinizing Weierstrass-elliptic function solutions and Jacobi-elliptic function solutions applicable to the CH equation. The methodology employed involves the utilization of the F-expansion method^15–17^. Our analysis encompasses the derivation of soliton-like solutions, Weierstrass-elliptic functions, and solutions characterized by hyperbolic and trigonometric functions within the framework of the cdCH equation. The mathematical expression governing the CH equation is presented according to the formula^19–21^

1 $$\begin{aligned} \begin{aligned} (N_{Cr})_t&= B_{Cr}(M_{N_{Cr}}-F_{Cr}(N_{Cr})_{xx}-K_{CrX}(N_x)_{xx})_{xx},\\ (N_{x})_t&= B_{x}(M_{N_{x}}-K_{x}(N_{Cr})_{xx}-F_{x}(N_x)_{xx})_{xx}, \end{aligned} \end{aligned}$$

where $N_x(x,t)$ and $N_{Cr}(x,t)$ represent the attractive regions of features *X* and *Cr* respectively, the system (1) takes on the following form

2 $$\begin{aligned} \begin{aligned} (N_{Cr})_t&=B_{Cr}[(M_{N^2_{Cr}}(N_{Cr})_{xx}+M_{N_{Cr}N_{x}}(N_x)_{xx}+2M_{N^2_{Cr}N_x}(N_{Cr})_x(N_{x})_x\\ {}&+ M_{N^3_{Cr}}((N_{Cr})_x)^2+M_{N_{Cr}N^2_{x}}((N_x)_x)^2-F_{Cr}(N_{Cr})_{xxx}-K_{CrX}(N_x)_{xxxx})],\\ (N_{x})_t&=B_{x}[(M_{N^2_{Cr}N_{x}}(N_{Cr})_{xx}+M_{N^2_{x}}(N_x)_{xx}+2M_{N^2_{Cr}N^2_x}(N_{Cr})_x(N_{x})_x\\ {}&+ M_{N^2_{Cr}N_{x}}((N_{Cr})_x)^2+M_{N_{Cr}N^3_{x}}((N_x)_x)^2-K_{xCr}(N_{Cr})_{xxx}-F_{x}(N_x)_{xxxx})]. \end{aligned} \end{aligned}$$

The regular solution model facilitates the expression of indigenous free energy through the following relation

3 $$\begin{aligned} \begin{aligned} M&=M^*(1-N_{Cr}-N_x)+M^{**}N_{Cr}+M^{***}N_{x}+\Theta _{FeX}N_{x}(1-N_{Cr}-N_x)\\ {}&+\Theta _{FeCr}N_{Cr}(1-N_{Cr}-N_x)+\Theta _{CrX}N_{Cr}N_{x}+RT[(1-N_{Cr}-N_x)\ln (1-N_{Cr}\\ {}&-N_x)+N_{Cr}\ln (N_{Cr})+N_x\ln (N_x)], \end{aligned} \end{aligned}$$

where the symbols $$M^{*}$$, $$M^{**}$$, and $$M^{***}$$ represent the energies associated with *Fe*,  *Cr*,  and *X* respectively, while $$\Theta _{FeCr}, \Theta _{FeX},$$ and $$\Theta _{CrX}$$ serve as integration parameters. Additionally, *T* denotes absolute temperature, and *R* stands for the gas constant. The formulation (3) is a direct outcome of this framework

4 $$\begin{aligned} \begin{aligned} M_{N^2_{Cr}}+2\Theta _{FeCr}-RT\bigg (\frac{1}{N_{Cr}}+\frac{1}{1-N_{Cr}-N_x} \bigg )&=0,\\ M_{N^2_{x}}+2\Theta _{FeX}-RT\bigg (\frac{1}{N_{x}}+\frac{1}{1-N_{Cr}-N_x} \bigg )&=0,\\ M_{N_{Cr}N_{x}}-\Theta _{CrX}+\Theta _{Fe\,Cr}+\Theta _{FeX}-\frac{Rt}{1-N_{Cr}-N_x}&=0. \end{aligned} \end{aligned}$$

The assessment of gradient energy and mobility entails employing the cdCH equation for binary iron alloys, exemplified by (Fe-Cr) and (Fe-X). Subsequently, these equations can undergo a linearization process for further analysis as

5 $$\begin{aligned} (N_i)_t+D_i(N_i)_{xx}+B_iF_i(N_i)_{xxxx}=0. \end{aligned}$$

The cdCH equation can be expressed as

6 $$\begin{aligned} \begin{aligned} \Psi _t&=\triangledown .B(\Psi )\triangledown [g(\Psi )-\epsilon ^2 \bigtriangleup \Psi ], (x,t)\in \Theta \times \Re ^+,\\ n.\triangledown \Psi&=n.B(\Psi )\triangledown [g(\Psi )-\epsilon ^2 \bigtriangleup \Psi ], (x,t)\in \partial \Theta \times \Re ^+. \end{aligned} \end{aligned}$$

Following Eq. (6), the cdCH equation can be formulated as follows

7 $$\begin{aligned} \Psi _t=\triangledown .[B(\Psi )\triangledown (g(\Psi )_{\Psi }-F \triangledown ^2\Psi )]. \end{aligned}$$

Here, $F(\Psi )$, $\Psi (x,t)$, and $g(\Psi )$ signify the mobility, attentiveness, and homogeneous free energy, respectively. Eq. (7) can be expressed as

8 $$\begin{aligned} \Psi _t+D^4\Psi =D^2A(\Psi )+\nu D\Psi ,\,\,\,\nu >0. \end{aligned}$$

In this context, the term $A(\Psi (x,t))$ signifies the chemical potential, $\Psi (x,t)$ denotes the concentration of one of the two phases in a system undergoing phase separation, and $\nu D(x,t)$ characterizes the phase transition influenced by the continuous fluid flow.

Utilizing the subsequent traveling wave transformation to Eq. (8) given by

9 $$\begin{aligned} \Psi (x,t)=W(\delta ), \,\,\, \delta =x-ct, \end{aligned}$$

with the wave velocity denoted as *c*, the result is the following ordinary differential equation (ODE)

10 $$\begin{aligned} cW'+W''''=(W^3-W)''+\nu W'. \end{aligned}$$

Twice integration of (10) reveals

11 $$\begin{aligned} \frac{1}{2}(c-1)W^2+W''-W^3+W=0. \end{aligned}$$

## An overview of the F-expansion method

To explore exact solutions for nonlinear evolution equations (NLEEs), we state an algorithm for the F-expansion method^15–18^. For a given nonlinear PDE with independent variables *x* and *t*, and dependent variable $\Psi$

12 $$\begin{aligned} \mathcal {U}(\Psi ,\Psi _t,\Psi _x,\Psi _{xx},\ldots )=0. \end{aligned}$$

Assuming $\Psi (x,t)=W(\delta )$, where the wave variable $\delta =x-ct$, the nonlinear PDE in Eq. (12) is thereby reduced to an ODE

13 $$\begin{aligned} \mathcal {N}(W,-c W',c^2 W'',-c^3 W''',\ldots )=0. \end{aligned}$$

Next, we seek solutions for the ODE in the following form

14 $$\begin{aligned} W(\delta )=\sum _{i=0}^{\phi } \eta _i G^i (\delta ). \end{aligned}$$

Here, $\eta _i$, (where $i=0,1,\,2,\ldots ,\phi$) represents constants to be determined, and $\phi$ is a positive integer that can be determined by balancing the nonlinear terms $W^3$ with linear term $W''$. Additionally, $G(\delta )$ satisfies the following auxiliary equation

15 $$\begin{aligned} W'(\delta )=\sigma \sqrt{XG^4(\delta )+YG^2(\delta )+Z}, \end{aligned}$$

where $\sigma =\pm 1$, and $X,\,Y,$ and *Z* are constants. Consequently, the last equation is satisfied for $G(\delta )$

16 $$\begin{aligned} \begin{aligned} G''&=2XG^3+YG,\\ G'''&=(6XG^2+Y)G'\\ G''''&=24X^2G^5+20XYG^3+(12XZ+Y^2)G\\&.\\&.\\&.. \end{aligned} \end{aligned}$$

In Tables 1 and 2, we present fifty two types of exact solutions for Eq. (15) (refer to^15–18^ for detailed information). Notably, these exact solutions can be employed to systematically construct additional exact solutions for Eq. (7).mobility, attentiveness, and homogeneous free

**Table 1:** The relationships delineating the values of $(X,\,Y,\,Z)$ and their respective $G(\delta )$ in Eq. (15) are established, where $X,\,Y$ and *Z* represent arbitrary constants, and $\varrho =\sqrt{1-\varrho ^2}$.

Case	*X*	*Y*	*Z*	$G(\delta )$
1	$\varrho ^2$	$-(1+\varrho ^2)$	1	${\text {sn}}\delta$
2	$\varrho ^2$	$-(1+\varrho ^2)$	1	${\text {cd}}\delta ={\text {cn}}\delta /{\text {dn}}\delta$
3	$-\varrho ^2$	$2\varrho ^2-1$	$1-\varrho ^2$	${\text {cn}}\delta$
4	$-1$	$2-\varrho ^2$	$\varrho ^2-1$	${\text {dn}}\delta$
5	1	$-(1+\varrho ^2)$	$\varrho ^2$	${\text {ns}}\delta =({\text {sn}}\delta )^{-1}$
6	1	$-(1+\varrho ^2)$	$\varrho ^2$	${\text {dc}}\delta ={\text {dn}}\delta /{\text {cn}}\delta$
7	$1-\varrho ^2$	$2\varrho ^2-1$	$-\varrho ^2$	${\text {nc}}\delta =({\text {cn}}\delta )^{-1}$
8	$\varrho ^2-1$	$2-\varrho ^2$	$-1$	${\text {nd}}\delta =({\text {dn}}\delta )^{-1}$
9	$1-\varrho ^2$	$2-\varrho ^2$	1	${\text {sc}}\delta ={\text {sn}}\delta /{\text {cn}}\delta$
10	$-\varrho ^2(1-\varrho ^2)$	$2\varrho ^2-1$	1	${\text {sd}}\delta ={\text {sn}}\delta /{\text {dn}}\delta$
11	1	$2-\varrho ^2$	$1-\varrho ^2$	${\text {cs}}\delta ={\text {cn}}\delta /{\text {sn}}\delta$
12	1	$2\varrho ^2-1$	$-\varrho ^2(1-\varrho ^2)$	${\text {ds}}\delta ={\text {dn}}\delta /{\text {sn}}\delta$
13	$\frac{1}{4}$	$(1-2\varrho ^2)/2$	$\frac{1}{4}$	${\text {ns}}\delta \pm {\text {cs}}\delta$
14	$(1-\varrho ^2)/4$	$(1-2\varrho ^2)/2$	$(1-\varrho ^2)/4$	${\text {nc}}\delta \pm {\text {sc}}\delta$
15	$\frac{1}{4}$	$(\varrho ^2-2)/2$	$\varrho ^2/4$	${\text {ns}}\delta \pm {\text {ds}}\delta$
16	$\varrho ^{2/4}$	$(\varrho ^2-2)/2$	$\varrho ^{2/4}$	${\text {sn}}\delta \pm i {\text {cn}}\delta$
17	$\varrho ^{2/4}$	$(\varrho ^2-2)/2$	$\varrho ^{2/4}$	$\sqrt{1-\varrho ^2}{\text {sd}}\delta \pm {\text {cd}}\delta$
18	1/4	$(1-\varrho ^2)/2$	1/4	$\varrho {\text {cd}}\delta \pm i\sqrt{1-\varrho ^2}{\text {nd}} \delta$
19	1/4	$(1-2\varrho ^2)/2$	1/4	$\varrho {\text {sn}}\delta \pm i{\text {dn}}\delta$
20	1/4	$(1-\varrho ^2)/2$	1/4	$\sqrt{1-\varrho ^2}{\text {sc}}\delta \pm {\text {dc}}\delta$
21	$(\varrho ^2-1)/4$	$(\varrho ^2+1)/2$	$(\varrho ^2-1)/4$	$\varrho {\text {sd}}\delta \pm {\text {nd}}\delta$
22	$\varrho ^{2/4}$	$(\varrho ^2-1)/2$	1/4	$\frac{{\text {sn}}\delta }{1\pm {\text {dn}}\delta }$
23	$-1/4$	$(\varrho ^2+1)/2$	$(1-\varrho ^2)^{2/4}$	$\varrho {\text {cn}} \delta \pm {\text {dn}}\delta$
24	$(1-\varrho ^2)^{2/4}$	$(\varrho ^2+1)/2$	1/4	${\text {ds}}\delta \pm {\text {cs}}\delta$
25	$\frac{\varrho ^4(1-\varrho ^2)}{2(2-\varrho ^2)}$	$\frac{2(1-\varrho ^2)}{\varrho ^2-2}$	$\frac{1-\varrho ^2}{2(2-\varrho ^2)}$	${\text {dc}}\delta \pm \sqrt{1-\varrho ^2}{\text {nc}}\delta$
26	$X>0$	$Y<0$	$\frac{\varrho ^2Y^2}{(\varrho ^2+1)^2 X}$	$\sqrt{\frac{-\varrho ^2Y}{(\varrho ^2+1)X}}{\text {sn}}\bigg (\sqrt{\frac{-Y}{\varrho ^2+1}}\delta \bigg )$
27	$X<0$	$Y>0$	$\frac{(1-\varrho ^2)Y^2}{(\varrho ^2-2)^2 X}$	$\sqrt{\frac{-Y}{(2-\varrho ^2)X}}{\text {dn}}\bigg (\sqrt{\frac{Y}{2-\varrho ^2}}\delta \bigg )$
28	$X<0$	$Y>0$	$\frac{\varrho ^2(\varrho ^2-1)Y^2}{(2\varrho ^2-1)^2 X}$	$\sqrt{-\frac{\varrho ^2Y}{(2\varrho ^2-1)X}}{\text {cn}}\bigg (\sqrt{\frac{Y}{2\varrho ^2-1}}\delta \bigg )$
29	1	$2-4\varrho ^2$	1	$\frac{{\text {sn}} \delta \,\,\, {\text {dn}} \delta }{{\text {cn}}\delta }$
30	$\varrho ^4$	2	1	$\frac{{\text {sn}} \delta \,\,\, {\text {cn}} \delta }{{\text {dn}}\delta }$
31	1	$\varrho ^2+2$	$1-2\varrho ^2+\varrho ^4$	$\frac{{\text {dn}} \delta \,\,\, {\text {cn}} \delta }{{\text {sn}}\delta }$
32	$\frac{A^2(\varrho -1)^2}{4}$	$\frac{\varrho ^2+1}{2}+3\varrho$	$\frac{(\varrho -1)^2}{4A^2}$	$\frac{{\text {dn}}\delta \,\,{\text {cn}}\delta }{A(1+{\text {sn}}\delta )(1+\varrho \,\,{\text {sn}}\delta )}$
33	$\frac{A^2(\varrho +1)^2}{4}$	$\frac{\varrho ^2+1}{2}-3\varrho$	$\frac{(\varrho +1)^2}{4A^2}$	$\frac{{\text {dn}}\delta \,\,{\text {cn}}\delta }{A(1+{\text {sn}}\delta )(1-\varrho \,\,{\text {sn}}\delta )}$
34	$-\frac{4}{\varrho }$	$6\varrho -\varrho ^2-1$	$-2\varrho ^3+\varrho ^4+\varrho ^2$	$\frac{\varrho \,{\text {cn}}\delta \,\,{\text {dn}}\delta }{\varrho \,\,{\text {sn}}^2+1}$
35	$\frac{4}{\varrho }$	$-6\varrho -\varrho ^2-1$	$2\varrho ^3+\varrho ^4+\varrho ^2$	$\frac{\varrho \,{\text {cn}}\delta \,\,{\text {dn}}\delta }{\varrho \,\,{\text {sn}}^2-1}$
36	1/4	$\frac{1-2\varrho ^2}{2}$	1/4	$\frac{{\text {sn}}}{1\pm {\text {cn}}\delta }$
37	$\frac{1-\varrho ^2}{4}$	$\frac{1+\varrho ^2}{2}$	$\frac{1-\varrho ^2}{4}$	$\frac{{\text {cn}}\delta }{1\pm {\text {sn}}\delta }$
38	$4\varrho _1$	$2+6\varrho _1-\varrho ^2$	$2+2\varrho _1-\varrho ^2$	$\frac{\varrho ^2\,{\text {sn}}\delta \,{\text {cn}}\delta }{\varrho _1-{\text {dn}}^2\delta }$
39	$-4\varrho _1$	$2-6\varrho _1-\varrho ^2$	$2-2\varrho _1-\varrho ^2$	$-\frac{\varrho ^2\,{\text {sn}}\delta \,{\text {cn}}\delta }{\varrho _1+{\text {dn}}^2\delta }$
40	$\frac{2-\varrho ^2-2\varrho _1}{4}$	$\frac{\varrho ^2}{2}-1-3\varrho _1$	$\frac{2-\varrho ^2-2\varrho _1}{4}$	$\frac{\varrho ^2 {\text {sn}}\delta \,\,{\text {cn}}\delta }{{\text {sn}}^2\delta +(1+\varrho _1){\text {dn}}\delta -1-\varrho _1}$
41	$\frac{2-\varrho ^2+2\varrho _1}{4}$	$\frac{\varrho ^2}{2}-1+3\varrho _1$	$\frac{2-\varrho ^2+2\varrho _1}{4}$	$\frac{\varrho ^2 {\text {sn}}\delta \,\,{\text {cn}}\delta }{{\text {sn}}^2\delta +(-1+\varrho _1){\text {dn}}\delta -1+\varrho _1}$
42	$\frac{C^2\varrho ^4-(B^2+C^2)\varrho ^2+B^2}{4}$	$\frac{\varrho ^2+1}{2}$	$\frac{\varrho ^2-1}{4(C^2\varrho ^2-B^2}$	$\frac{\sqrt{\frac{(B^2-C^2)}{(B^2-C^2\varrho ^2)}}+{\text {sn}}\delta }{B\,{\text {cn}}\delta +C\,\,{\text {dn}}\delta }$
43	$\frac{B^2+C^2\varrho ^2}{4}$	$\frac{1}{2}-\varrho ^2$	$\frac{1}{4(C^2\varrho ^2+B^2)}$	$\frac{\sqrt{\frac{(B^2+C^2\varrho ^2-C^2)}{(B^2+C^2\varrho ^2)}}+{\text {cn}}\delta }{B\,{\text {sn}}\delta +C\,\,{\text {dn}}\delta }$
44	$\frac{B^2+C^2}{4}$	$\frac{\varrho ^2}{2}-1$	$\frac{\varrho ^4}{4(C^2+B^2)}$	$\frac{\sqrt{\frac{(B^2-C^2\varrho ^2+C^2)}{(B^2+C^2)}}+{\text {dn}}\delta }{B\,{\text {sn}}\delta +C\,\,{\text {cn}}\delta }$
45	$-(\varrho ^2+2\varrho +1)B^2$	$2\varrho ^2+2$	$\frac{2\varrho -\varrho ^2-1}{B^2}$	$\frac{\varrho \,{\text {sn}}^2\delta -1}{B(\varrho \,{\text {sn}}^2\delta +1)}$
46	$-(\varrho ^2-2\varrho +1)B^2$	$2\varrho ^2+2$	$-\frac{2\varrho +\varrho ^2+1}{B^2}$	$\frac{\varrho \,{\text {sn}}^2\delta +1}{B(\varrho \,{\text {sn}}^2\delta -1)}$

**Table 2:** Weierstrass-elliptic function solutions for Eq. (15), where $D=\frac{1}{2}\big (-5Y\pm \sqrt{9Y^2-36XZ}\big )$ and $\wp (\delta ; f_2,f_3)=\frac{d\wp (\delta ; f_2,f_3)}{d\delta }$.

Case	$f_2$	$f_3$	$G(\delta )$
47	$\frac{4}{3}(Y^2-3XZ)$	$\frac{4Y}{27}(-2Y^2+9XZ)$	$\sqrt{\frac{1}{X}(\wp (\delta ; f_2,f_3)-\frac{1}{3}Y)}$
48	$\frac{4}{3}(Y^2-3XZ)$	$\frac{4Y}{27}(-2Y^2+9XZ)$	$\sqrt{\frac{3Z}{3\wp ((\delta ; f_2,f_3)-Y)}}$
49	$\frac{-(5YD+4Y^2+33XYZ)}{12}$	$\frac{21Y^2D-63XZD+20Y^3-27XYZ}{216}$	$\frac{\sqrt{12Z\wp (\delta ; f_2,f_3)+2Z(2Y+D)}}{12\wp (\delta ; f_2,f_3)+D}$
50	$\frac{1}{12}Y^2+XZ$	$\frac{1}{216}Y(36XZ-Y^2)$	$\frac{\sqrt{Z}[6\wp (\delta ; f_2,f_3)+Y]}{3\wp '(\delta ; f_2,f_3)}$
51	$\frac{1}{12}Y^2+XZ$	$\frac{1}{216}Y(36XZ-Y^2)$	$\frac{3\wp '(\delta ; f_2,f_3)}{\sqrt{X}[6\wp (\delta ; f_2,f_3)+Y]}$
52	$\frac{2Y^2}{9}$	$\frac{Y^3}{54}$	$\frac{Y\sqrt{-15Y/2X}\wp (\delta ; f_2,f_3)}{3\wp (\delta ; f_2,f_3)+Y},\,\,\,Z=\frac{5Y^2}{36X}$

## Exact Jacobi-elliptic function solutions

Balancing the nonlinear term $W^3$ with the linear term $W''$ results in $\phi =1$. Thus, based on Eq. (14), we can make the following selection

17 $$\begin{aligned} W(\delta )=\eta _0+\eta _1 G(\delta ). \end{aligned}$$

where $\eta _0$ and $\eta _1$ represent undetermined constants. By substituting Eqs. (17) and (15) into Eq. (7) and subsequently equating the coefficients of $G^j(\delta )G'(\delta )$, $j=0,1,2,\ldots ,5,$ a system of algebraic equations for $\eta _0,\,\,\eta _1,~\eta _2$ and $\alpha$ is followed

18 $$\begin{aligned} \begin{aligned} \eta _0^2\eta _1-2\alpha ^2 Z\eta _1\eta _2+\alpha ^2Y \eta _0\eta _1&=0,\\ 2\eta _0\eta _1^2+2\eta _0^2\eta _2-4\alpha ^2 Z \eta _2^2+8\alpha ^2Y\eta _0\eta _2&=0,\\ \eta _1^3+6\eta _0\eta _1\eta _2+3\alpha ^2Y\eta _1\eta _2+6\alpha ^2 X\eta _0\eta _1&=0,\\ 4\eta _1^2\eta _2+4\eta _0\eta _2^2+4\alpha ^2X\eta _1^2+24\alpha ^2X\eta _0\eta _2&=0,\\ 5\eta _1\eta _2^2+20\alpha ^2X\eta _1\eta _2&=0,\\ 2\eta _2^3+12\alpha ^2X\eta _2^2&=0. \end{aligned} \end{aligned}$$

Upon solving the aforementioned overdetermined system with the assistance of *Mathematica*, the solutions are determined as follows

19 $$\begin{aligned} \eta _0=\pm \frac{1}{\sqrt{2}}i,~~~~~~~~~~~~~\,\,\,\eta _1=\eta _1. \end{aligned}$$

Substituting these results into Eq. (17), we have the following formal solution of Eq. (7)

20 $$\begin{aligned} W(\delta )=\pm \frac{1}{\sqrt{2}}i+\eta _1 G(\delta ),\,\,\,\text {where}\,\,\delta =x-ct. \end{aligned}$$

Utilizing Table 1 and the formal solution (20), it becomes possible to deduce more comprehensive combined Jacobian-elliptic function solutions for Eq. (7). Consequently, the following exact solutions are derived.

Class 1: $$X=\varrho ^2,\,\,Y=-(1+\varrho ^2),\,\,Z=1,\,\,G(\delta )={\text {sn}}\delta ,$$

21 $$\begin{aligned} \Psi _1(x,t)=\pm \frac{1}{\sqrt{2}}i+\eta _1{\text {sn}}(x-ct). \end{aligned}$$

Class 2: $$X=\varrho ^2,\,\,Y=-(1+\varrho ^2),\,\,Z=1,\,\,G(\delta )={\text {cd}}\delta ,$$

22 $$\begin{aligned} \Psi _2(x,t)=\pm \frac{1}{\sqrt{2}}i+\eta _1{\text {cd}}(x-ct). \end{aligned}$$

Class 3: $$X=-\varrho ^2,\,\,Y=2\varrho ^2-1,\,\,Z=1-\varrho ^2,\,\,G(\delta )={\text {cn}}\delta ,$$

23 $$\begin{aligned} \Psi _3(x,t)=\pm \frac{1}{\sqrt{2}}i+\eta _1{\text {cn}}(x-ct). \end{aligned}$$

Class 4: $$X=-1,\,\,Y=2-\varrho ^2,\,\,Z=\varrho ^2-1,\,\,G(\delta )={\text {dn}}\delta$$

24 $$\begin{aligned} \Psi _4(x,t)=\pm \frac{1}{\sqrt{2}}i+\eta _1{\text {dn}}(x-ct). \end{aligned}$$

Class 5: $$X=1,\,\,Y=-(1+\varrho ^2),\,\,Z=\varrho ^2,\,\,G(\delta )={\text {ns}}\delta ,$$

25 $$\begin{aligned} \Psi _5(x,t)=\pm \frac{1}{\sqrt{2}}i+\eta _1{\text {ns}}(x-ct). \end{aligned}$$

Class 6: $$X=1,\,\,Y=-(1+\varrho ^2),\,\,Z=\varrho ^2,\,\,G(\delta )={\text {dc}}\delta ,$$

26 $$\begin{aligned} \Psi _6(x,t)=\pm \frac{1}{\sqrt{2}}i+\eta _1{\text {dc}}(x-ct). \end{aligned}$$

Class 7: $$X=1-\varrho ^2,\,\,Y=2\varrho ^2-1,\,\,Z=-\varrho ^2,\,\,G(\delta )={\text {nc}}\delta ,$$

27 $$\begin{aligned} \Psi _7(x,t)=\pm \frac{1}{\sqrt{2}}i+\eta _1{\text {nc}}(x-ct). \end{aligned}$$

Class 8: $$X=\varrho ^2-1,\,\,Y=2-\varrho ^2,\,\,Z=-1,\,\,G(\delta )={\text {nd}}\delta ,$$

28 $$\begin{aligned} \Psi _8(x,t)=\pm \frac{1}{\sqrt{2}}i+\eta _1{\text {nd}}(x-ct). \end{aligned}$$

Class 9: $$X=1-\varrho ^2,\,\,Y=2-\varrho ^2,\,\,Z=1,\,\,G(\delta )={\text {sc}}\delta ,$$

29 $$\begin{aligned} \Psi _9(x,t)=\pm \frac{1}{\sqrt{2}}i+\eta _1{\text {sc}}(x-ct). \end{aligned}$$

Class 10: $$X=-\varrho ^2(1-\varrho ^2),\,\,Y=2\varrho ^2-1,\,\,Z=1,\,\,G(\delta )={\text {sd}}\delta ,$$

30 $$\begin{aligned} \Psi _{10}(x,t)=\pm \frac{1}{\sqrt{2}}i+\eta _1{\text {sd}}(x-ct). \end{aligned}$$

Class 11: $$X=1,\,\,Y=2-\varrho ^2,\,\,Z=1-\varrho ^2,\,\,G(\delta )={\text {cs}}\delta ,$$

31 $$\begin{aligned} \Psi _{11}(x,t)=\pm \frac{1}{\sqrt{2}}i+\eta _1{\text {cs}}(x-ct). \end{aligned}$$

Class 12: $$X=1,\,\,Y=2\varrho ^2-1,\,\,Z=-\varrho ^2(1-\varrho ^2),\,\,G(\delta )={\text {ds}}\delta ,$$

32 $$\begin{aligned} \Psi _{12}(x,t)=\pm \frac{1}{\sqrt{2}}i+\eta _1{\text {ds}}(x-ct). \end{aligned}$$

Class 13: $$X=1/4,\,\,Y=(1-2\varrho ^2)/2,\,\,Z=1/4,\,\,G(\delta )={\text {ns}}\delta \pm {\text {cs}}\delta ,$$

33 $$\begin{aligned} \Psi _{13}(x,t)=\pm \frac{1}{\sqrt{2}}i+\eta _1({\text {ns}}(x-ct)\pm {\text {cs}}(x-ct)). \end{aligned}$$

Class 14: $$X=(1-\varrho ^2)/4,\,\,Y=(1+\varrho ^2)/2,\,\,Z=(1-\varrho ^2)/4,\,\,G(\delta )={\text {nc}}\delta \pm {\text {sc}}\delta ,$$

34 $$\begin{aligned} \Psi _{14}(x,t)=\pm \frac{1}{\sqrt{2}}i+\eta _1({\text {nc}}(x-ct)\pm {\text {sc}}(x-ct)). \end{aligned}$$

Class 15: $$X=1/4,\,\,Y=(\varrho ^2-2)/2,\,\,Z=\varrho ^2/4,\,\,G(\delta )={\text {ns}}\delta \pm {\text {ds}}\delta ,$$

35 $$\begin{aligned} \Psi _{15}(x,t)=\pm \frac{1}{\sqrt{2}}i+\eta _1({\text {ns}}(x-ct)\pm {\text {ds}}(x-ct)). \end{aligned}$$

Class 16: $$X=\varrho ^2/4,\,\,Y=(\varrho ^2-2)/2,\,\,Z=\varrho ^2/4,\,\,G(\delta )={\text {sn}}\delta \pm i\,{\text {cn}}\delta ,$$

36 $$\begin{aligned} \Psi _{16}(x,t)=\pm \frac{1}{\sqrt{2}}i+\eta _1({\text {sn}}(x-ct)\pm i\,{\text {cn}}(x-ct)). \end{aligned}$$

Class 17: $$X=\varrho ^2/4,\,\,Y=(\varrho ^2-2)/2,\,\,Z=\varrho ^2/4,\,\,G(\delta )=\sqrt{1-\varrho ^2}{\text {sd}}\delta \pm {\text {cd}}\delta ,$$

37 $$\begin{aligned} \Psi _{17}(x,t)=\pm \frac{1}{\sqrt{2}}i+\eta _1(\sqrt{1-\varrho ^2}{\text {sd}}(x-ct)\pm {\text {cd}}(x-ct)). \end{aligned}$$

Class 18: $$X=1/4,\,\,Y=(1-\varrho ^2)/2,\,\,Z=1/4,\,\,G(\delta )=\varrho \,{\text {cd}}\delta \pm i\,\sqrt{1-\varrho ^2}{\text {nd}}\delta ,$$

38 $$\begin{aligned} \Psi _{18}(x,t)=\pm \frac{1}{\sqrt{2}}i+\eta _1(\varrho \,{\text {cd}}(x-ct)\pm i\,\sqrt{1-\varrho ^2}{\text {nd}}(x-ct)). \end{aligned}$$

Class 19: $$X=1/4,\,\,Y=(1-2\varrho ^2)/2,\,\,Z=1/4,\,\,G(\delta )=\varrho \,{\text {sn}}\delta \pm i\,{\text {dn}}\delta ,$$

39 $$\begin{aligned} \Psi _{19}(x,t)=\pm \frac{1}{\sqrt{2}}i+\eta _1(\varrho \,{\text {sn}}(x-ct)\pm i\,{\text {dn}}(x-ct)). \end{aligned}$$

Class 20: $$X=1/4,\,\,Y=(1-\varrho ^2)/2,\,\,Z=1/4,\,\,G(\delta )=\sqrt{1-\varrho ^2}{\text {sc}}\delta \pm i\,{\text {dc}}\delta ,$$

40 $$\begin{aligned} \Psi _{20}(x,t)=\pm \frac{1}{\sqrt{2}}i+\eta _1(\sqrt{1-\varrho ^2}{\text {sc}}(x-ct)\pm i\,{\text {dc}}(x-ct)). \end{aligned}$$

Class 21: $$X=(\varrho ^2-1)/4,\,\,Y=(\varrho ^2+1)/2,\,\,Z=(\varrho ^2-1)/4,\,\,G(\delta )=\varrho \,{\text {sd}}\delta \pm {\text {nd}}\delta ,$$

41 $$\begin{aligned} \Psi _{21}(x,t)=\pm \frac{1}{\sqrt{2}}i+\eta _1(\varrho \,{\text {sd}}(x-ct)\pm {\text {nd}}(x-ct)). \end{aligned}$$

Class 22: $$X=\varrho ^2/4,\,\,Y=(\varrho ^2-2)/2,\,\,Z=1/4,\,\,G(\delta )=\frac{{\text {sn}}\delta }{1\pm {\text {dn}}\delta },$$

42 $$\begin{aligned} \Psi _{22}(x,t)=\pm \frac{1}{\sqrt{2}}i+\eta _1\frac{{\text {sn}}(x-ct)}{1\pm {\text {dn}}(x-ct)}. \end{aligned}$$

Class 23: $$X=-1/4,\,\,Y=(\varrho ^2+1)/2,\,\,Z=(1-\varrho ^2)^2/4,\,\,G(\delta )=\varrho \,{\text {cn}}\delta \pm {\text {dn}}\delta 
,$$

43 $$\begin{aligned} \Psi _{23}(x,t)=\pm \frac{1}{\sqrt{2}}i+\eta _1(\varrho \,{\text {cn}}(x-ct)\pm {\text {dn}}(x-ct)). \end{aligned}$$

Class 24: $$X=(1-\varrho ^2)^2/4,\,\,Y=(\varrho ^2+1)/2,\,\,Z=1/4,\,\,G(\delta )={\text {ds}}\delta \pm {\text {cs}}\delta ,$$

44 $$\begin{aligned} \Psi _{24}(x,t)=\pm \frac{1}{\sqrt{2}}i+\eta _1({\text {ds}}(x-ct)\pm {\text {cs}}(x-ct)). \end{aligned}$$

Class 25: $$X=\frac{\varrho ^4(1-\varrho ^2)}{2(2-\varrho ^2)},\,\,Y=\frac{2(1-\varrho ^2)}{\varrho ^2-2},\,\,Z=\frac{1-\varrho ^2}{\varrho ^2-1},\,\,G(\delta )={\text {dc}}\delta \pm \sqrt{1-\varrho ^2}{\text {nc}}\delta ,$$

45 $$\begin{aligned} \Psi _{25}(x,t)=\pm \frac{1}{\sqrt{2}}i+\eta _1({\text {dc}}(x-ct)\pm \sqrt{1-\varrho ^2}{\text {nc}}(x-ct)). \end{aligned}$$

Class 26: $$Z=\frac{\varrho ^2Y^2}{(\varrho ^2+1)^2X},\,\,Y<0,\,\,X>0,\,\,G(\delta )=\sqrt{\frac{-\varrho ^2Y}{(\varrho ^2+1)X}}{\text {sn}}\bigg (\sqrt{\frac{-Y}{\varrho ^2+1}}\delta \bigg ),$$

46 $$\begin{aligned} \Psi _{26}(x,t)=\pm \frac{1}{\sqrt{2}}i+\eta _1\sqrt{\frac{-\varrho ^2Y}{(\varrho ^2+1)X}}{\text {sn}}\bigg (\sqrt{\frac{-Y}{\varrho ^2+1}}(x-ct)\bigg ). \end{aligned}$$

Class 27: $$Z=\frac{(1-\varrho ^2)Y^2}{(\varrho ^2-2)^2X},\,\,Y>0,\,\,X<0,\,\,G(\delta )=\sqrt{\frac{Y}{(2-\varrho ^2)X}}{\text {dn}}\bigg (\sqrt{\frac{Y}{2-\varrho ^2}}\delta \bigg ),$$

47 $$\begin{aligned} \Psi _{27}(x,t)=\pm \frac{1}{\sqrt{2}}i+\eta _1\sqrt{\frac{Y}{(2-\varrho ^2)X}}{\text {dn}}\bigg (\sqrt{\frac{-Y}{2-\varrho ^2}}(x-ct)\bigg ). \end{aligned}$$

Class 28: $$Z=\frac{\varrho ^2(\varrho ^2-1)Y^2}{(2\varrho ^2-1)^2X},\,\,Y>0,\,\,X<0,\,\,G(\delta )=\sqrt{\frac{-\varrho ^2Y}{(2\varrho ^2-1)X}}{\text {cn}} \bigg (\sqrt{\frac{Y}{2\varrho ^2-1}}\delta \bigg ),$$

48 $$\begin{aligned} \Psi _{28}(x,t)=\pm \frac{1}{\sqrt{2}}i+\eta _1\sqrt{\frac{-\varrho ^2Y}{(2\varrho ^2-1)X}}{\text {cn}}\bigg (\sqrt{\frac{Y}{2\varrho ^2-1}}(x-ct)\bigg ). \end{aligned}$$

Class 29: $$X=1,\,\,Y=2-4\varrho ^2,\,\,Z=1,\,\,G(\delta )=\frac{{\text {sn}}\delta \,{\text {dn}}\delta }{{\text {cn}}\delta },$$

49 $$\begin{aligned} \Psi _{29}(x,t)=\pm \frac{1}{\sqrt{2}}i+\eta _1\frac{{\text {sn}}(x-ct)\,{\text {dn}}(x-ct)}{{\text {cn}}(x-ct)}. \end{aligned}$$

Class 30: $$X=\varrho ^2,\,\,Y=2,\,\,Z=1,\,\,G(\delta )=\frac{{\text {sn}}\delta \,{\text {cn}}\delta }{{\text {dn}}\delta },$$

50 $$\begin{aligned} \Psi _{30}(x,t)=\pm \frac{1}{\sqrt{2}}i+\eta _1\frac{{\text {sn}}(x-ct)\,{\text {cn}}(x-ct)}{{\text {dn}}(x-ct)}. \end{aligned}$$

Class 31: $$X=1,\,\,Y=\varrho ^2+2,\,\,Z=1-2\varrho ^2+\varrho ^4,\,\,G(\delta )=\frac{{\text {cn}}\delta \,{\text {dn}}\delta }{{\text {sn}}\delta },$$

51 $$\begin{aligned} \Psi _{31}(x,t)=\pm \frac{1}{\sqrt{2}}i+\eta _1\frac{{\text {cn}}(x-ct)\,{\text {dn}}(x-ct)}{{\text {sn}}(x-ct)}. \end{aligned}$$

Class 32: $$X=\frac{A^2(\varrho -1)^2}{4},\,\,Y=\frac{\varrho ^2+1}{2},\,\,Z=\frac{(\varrho -1)^2}{4A^2},\,\,G(\delta )=\frac{{\text {dn}}\delta \, {\text {cn}}\delta }{A(1+{\text {sn}}\delta )(1+\varrho \,{\text {sn}}\delta )},$$

52 $$\begin{aligned} \Psi _{32}(x,t)=\pm \frac{1}{\sqrt{2}}i+\eta _1\frac{{\text {dn}}(x-ct)\,{\text {cn}}(x-ct)}{A(1+{\text {sn}}(x-ct))(1+\varrho \,{\text {sn}}(x-ct))}. \end{aligned}$$

Class 33: $$X=\frac{A^2(\varrho +1)^2}{4},\,\,Y=\frac{\varrho ^2+1}{2}-3\varrho ,\,\,Z=\frac{(\varrho +1)^2}{4A^2},\,\,G(\delta )=\frac{{\text {dn}}\delta \, {\text {cn}}\delta }{A(1+{\text {sn}}\delta )(1-\varrho \,{\text {sn}}\delta )},$$

53 $$\begin{aligned} \Psi _{33}(x,t)=\pm \frac{1}{\sqrt{2}}i+\eta _1\frac{{\text {dn}}(x-ct)\,{\text {cn}}(x-ct)}{A(1+{\text {sn}}(x-ct)) (1-\varrho \,{\text {sn}}(x-ct))}. \end{aligned}$$

Class 34: $$X=\frac{-4}{\varrho },\,\,Y=6\varrho -\varrho ^2-1,\,\,Z=-2\varrho ^3+\varrho ^4+\varrho ^2,\,\,G(\delta )=\frac{\varrho \,{\text {cn}}\delta \,{\text {dn}}\delta }{\varrho \,{\text {sn}}^2\delta +1},$$

54 $$\begin{aligned} \Psi _{34}(x,t)=\pm \frac{1}{\sqrt{2}}i+\eta _1\frac{\varrho \,{\text {cn}}(x-ct)\,{\text {dn}}(x-ct)}{\varrho \,{\text {sn}}^2(x-ct)+1}. \end{aligned}$$

Class 35:$$X=\frac{4}{\varrho },\,\,Y=-6\varrho -\varrho ^2-1,\,\,Z=2\varrho ^3+\varrho ^4+\varrho ^2,\,\,G(\delta )=\frac{\varrho \,{\text {cn}}\delta \,{\text {dn}}\delta }{\varrho \,{\text {sn}}^2\delta -1},$$

55 $$\begin{aligned} \Psi _{35}(x,t)=\pm \frac{1}{\sqrt{2}}i+\eta _1\frac{\varrho \,{\text {cn}}(x-ct)\,{\text {dn}}(x-ct)}{\varrho \,{\text {sn}}^2(x-ct)-1}. \end{aligned}$$

Class 36: $$X=1/4,\,\,Y=\frac{1-2\varrho ^2}{2},\,\,Z=1/4,\,\,G(\delta )=\frac{{\text {sn}}\delta }{1\pm {\text {cn}}\delta },$$

56 $$\begin{aligned} \Psi _{36}(x,t)=\pm \frac{1}{\sqrt{2}}i+\eta _1\frac{{\text {sn}}(x-ct)}{1\pm {\text {cn}}(x-ct)}. \end{aligned}$$

Class 37: $$X=\frac{1-\varrho ^2}{4},\,\,Y=\frac{1+\varrho ^2}{2},\,\,Z=\frac{1-\varrho ^2}{4},\,\,G(\delta )=\frac{{\text {cn}}\delta }{1\pm {\text {sn}}\delta },$$

57 $$\begin{aligned} \Psi _{37}(x,t)=\pm \frac{1}{\sqrt{2}}i+\eta _1\frac{{\text {cn}}(x-ct)}{1\pm {\text {sn}}(x-ct)}. \end{aligned}$$

**Class 38:** $$X=4\varrho _1,\,\,Y=2+6\varrho _1-\varrho ^2,\,\,Z=2+2\varrho _1-\varrho ^2,\,\,G(\delta )=\frac{\varrho ^2\,{\text {sn}}\delta \,{\text {cn}}\delta }{\varrho _1-{\text {dn}}^2\delta },$$

58 $$\begin{aligned} \Psi _{38}(x,t)=\pm \frac{1}{\sqrt{2}}i+\eta _1\frac{\varrho ^2\,{\text {sn}}(x-ct)\,{\text {cn}}(x-ct)}{\varrho _1-{\text {dn}}^2(x-ct)}. \end{aligned}$$

Class 39: $$X=-4\varrho _1,\,\,Y=2-6\varrho _1-\varrho ^2,\,\,Z=2-2\varrho _1-\varrho ^2,\,\,G(\delta )=\frac{-\varrho ^2\,{\text {sn}}\delta \,{\text {cn}}\delta }{\varrho _1+{\text {dn}}^2\delta },$$

59 $$\begin{aligned} \Psi _{39}(x,t)=\pm \frac{1}{\sqrt{2}}i+\eta _1\frac{-\varrho ^2\,{\text {sn}}(x-ct)\,{\text {cn}}(x-ct)}{\varrho _1+{\text {dn}}^2(x-ct)}. \end{aligned}$$

Class 40: $$X=\frac{2-\varrho ^2-2\varrho _1}{4},\,\,Y=\frac{\varrho ^2}{2}-1-3\varrho _1,\,\,Z=\frac{2-\varrho ^2-2\varrho _1}{4},\,\,G(\delta )=\frac{\varrho ^2{\text {sn}}\delta \,{\text {cn}} \delta }{{\text {sn}}^2\delta +(1+\varrho _1){\text {dn}}\delta -1-\varrho _1},$$

60 $$\begin{aligned} \Psi _{40}(x,t)=\pm \frac{1}{\sqrt{2}}i+\eta _1\frac{\varrho ^2{\text {sn}}(x-ct) \,{\text {cn}} (x-ct)}{{\text {sn}}^2(x-ct)+(1+\varrho _1){\text {dn}}(x-ct) -1-\varrho _1}. \end{aligned}$$

Class 41: $$X=\frac{2-\varrho ^2+2\varrho _1}{4},\,\,Y=\frac{\varrho ^2}{2}-1+3\varrho _1,\,\,Z=\frac{2-\varrho ^2+2\varrho _1}{4},\,\,G(\delta )=\frac{\varrho ^2{\text {sn}}\delta \,{\text {cn}} \delta }{{\text {sn}}^2\delta +(-1+\varrho _1){\text {dn}}\delta -1-\varrho _1},$$

61 $$\begin{aligned} \Psi _{41}(x,t)=\pm \frac{1}{\sqrt{2}}i+\eta _1\frac{\varrho ^2{\text {sn}}(x-ct) \,{\text {cn}} (x-ct)}{{\text {sn}}^2(x-ct)+(-1+\varrho _1){\text {dn}}(x-ct) -1-\varrho _1}. \end{aligned}$$

Class 42: $$X=\frac{C^2\varrho ^4-(B^2+C^2)\varrho ^2+B^2}{4},\,\,Y=\frac{\varrho ^2+1}{2},\,\,Z=\frac{\varrho ^2-1}{4(C^2\varrho ^2-B^2)},\,\, G(\delta )=\frac{\sqrt{\frac{B^2-C^2}{B^2-C^2\varrho ^2}}+{\text {sn}}\delta }{B\,{\text {cn}}\delta +C\,\,{\text {dn}}\delta }.$$

62 $$\begin{aligned} \Psi _{42}(x,t)=\pm \frac{1}{\sqrt{2}}i+\eta _1\frac{\sqrt{\frac{B^2-C^2}{B^2-C^2\varrho ^2}}+{\text {sn}}(x-ct)}{B\,{\text {cn}}(x-ct)+C\,\,{\text {dn}}(x-ct)}. \end{aligned}$$

Class 43: $$X=\frac{B^2+C^2\varrho ^2}{4},\,\,Y=\frac{1}{2}-\varrho ^2,\,\,Z=\frac{1}{4(C^2\varrho ^2+B^2)},\,\, G(\delta )=\frac{\sqrt{\frac{B^2+C^2\varrho ^2-C^2}{B^2+C^2\varrho ^2}}+{\text {cn}}\delta }{B\,{\text {sn}}\delta +C\,\,{\text {dn}}\delta }$$.

63 $$\begin{aligned} \Psi _{43}(x,t)=\pm \frac{1}{\sqrt{2}}i+\eta _1\frac{\sqrt{\frac{B^2+C^2\varrho ^2-C^2}{B^2+C^2\varrho ^2}}+{\text {cn}}(x-ct)}{B\,{\text {sn}}(x-ct)+C\,\,{\text {dn}}(x-ct)}. \end{aligned}$$

Class 44: $$X=\frac{B^2+C^2}{4} ,\,\,Y=\frac{\varrho ^2}{2}-1,\,\,Z=\frac{\varrho ^4}{4(C^2+B^2)},\,\,G(\delta )=\frac{\sqrt{\frac{B^2+C^2-C^2\varrho ^2}{B^2+C^2}}+ {\text {dn}}\delta }{B\,{\text {sn}}\delta +C\,\,{\text {cn}}\delta },$$

64 $$\begin{aligned} \Psi _{44}(x,t)=\pm \frac{1}{\sqrt{2}}i+\eta _1\frac{\sqrt{\frac{B^2+C^2-C^2\varrho ^2}{B^2+C^2}}+{\text {dn}}(x-ct)}{B\,{\text {sn}}(x-ct)+C\,\,{\text {cn}}(x-ct)}. \end{aligned}$$

Class 45: $$X=-(\varrho ^2+2\varrho +1)B^2,\,\,Y=2\varrho ^2+2,\,\,Z=\frac{2\varrho -\varrho ^2-1}{B^2},\,\, G(\delta )=\frac{\varrho \,{\text {sn}}^2\delta -1}{B(\varrho \,{\text {sn}}^2\delta +1)},$$

65 $$\begin{aligned} \Psi _{45}(x,t)=\pm \frac{1}{\sqrt{2}}i+\eta _1\frac{\varrho \,{\text {sn}}^2(x-ct)-1}{B(\varrho \,{\text {sn}}^2(x-ct)+1)}. \end{aligned}$$

Class 46: $$X=-(\varrho ^2-2\varrho +1)B^2,\,\,Y=2\varrho ^2+2,\,\,Z=-\frac{2\varrho +\varrho ^2+1}{B^2},\,\, G(\delta )=\frac{\varrho \,{\text {sn}}^2\delta +1}{B(\varrho \,{\text {sn}}^2\delta -1)},$$

66 $$\begin{aligned} \Psi _{46}(x,t)=\pm \frac{1}{\sqrt{2}}i+\eta _1\frac{\varrho \,{\text {sn}}^2(x-ct)+1}{B(\varrho \,{\text {sn}}^2(x-ct)-1)}. \end{aligned}$$

## Weiestrass-elliptic function solutions

By incorporating the solutions provided in^22^, as outlined in Table 2, and utilizing Eq. (20), the resulting set of exact solutions is as follows; Class 47: $f_2=\frac{4}{3}(Y^2-3XZ),\,\,f_3=\frac{4Y}{27}(-2Y^2+9XZ),\,\,G(\delta )=\sqrt{\frac{1}{X}(\wp (\delta ; f_2,f_3)-\frac{1}{3}Y)},$

67 $$\begin{aligned} \Psi _{47}(x,t)=\pm \frac{1}{\sqrt{2}}i+\eta _1\sqrt{\frac{1}{X}(\wp (x-ct; f_2,f_3)-\frac{1}{3}Y)}. \end{aligned}$$

Class 48: $$f_2=\frac{4}{3}(Y^2-3XZ),\,\,f_3=\frac{4Y}{27}(-2Y^2+9XZ),\,\,G(\delta )=\sqrt{\frac{3Z}{3\wp (\delta ; f_2,f_3)-Y}},$$

68 $$\begin{aligned} \Psi _{48}(x,t)=\pm \frac{1}{\sqrt{2}}i+\eta _1\sqrt{\frac{3Z}{3\wp (x-ct; f_2,f_3)-Y}}. \end{aligned}$$

Class 49: $$f_2=\frac{-(5YD+4Y^2+33XYZ)}{12},\,\,f_3=\frac{21Y^2D-63XZD+20Y^3-27XYZ}{216},\,\, G(\delta )=\frac{\sqrt{12Z\wp (\delta ; f_2,f_3)+2Z(2Y+D)}}{12\wp (x-ct; f_2,f_3)+D},$$

69 $$\begin{aligned} \Psi _{49}(x,t)=\pm \frac{1}{\sqrt{2}}i+\eta _1\frac{\sqrt{12Z\wp (x-ct; f_2,f_3)+2Z(2Y+D)}}{12\wp (x+ct; f_2,f_3)+D}. \end{aligned}$$

Class 50: $$f_2=\frac{1}{12}Y^2+XZ,\,\,f_3=\frac{1}{216}Y(36XZ-Y^2),\,\,G(\delta )=\frac{\sqrt{Z}[6\wp (\delta ; f_2,f_3)+Y]}{3\wp '(\delta ; f_2,f_3)},$$

70 $$\begin{aligned} \Psi _{50}(x,t)=\pm \frac{1}{\sqrt{2}}i+\eta _1\frac{\sqrt{Z}[6\wp (x-ct; f_2,f_3)+Y]}{3\wp '(x-ct; f_2,f_3)}. \end{aligned}$$

Class 51: $$f_2=\frac{1}{12}Y^2+XZ,\,\,f_3=\frac{1}{216}Y(36XZ-Y^2),\,\,G(\delta )=\frac{3\wp '(\delta ; f_2,f_3)}{\sqrt{X}[6\wp (\delta ; f_2,f_3)+Y]},$$

71 $$\begin{aligned} \Psi _{51}(x,t)=\pm \frac{1}{\sqrt{2}}i+\eta _1\frac{3\wp '(x-ct; f_2,f_3)}{\sqrt{X}[6\wp (x-ct; f_2,f_3)+Y]}. \end{aligned}$$

Class 52: $$f_2=\frac{2Y^2}{9},\,\,f_3=\frac{Y^3}{54},\,\,G(\delta )=\frac{Y\sqrt{-15Y/2X}\wp (\delta ; f_2,f_3)}{3\wp (\delta ; f_2,f_3)+Y},\,\,\,Z=\frac{5Y^2}{36X},$$

72 $$\begin{aligned} \Psi _{52}(x,t)=\pm \frac{1}{\sqrt{2}}i+\eta _1\frac{Y\sqrt{-15Y/2X}\wp (x-ct; f_2,f_3)}{3\wp (x-ct; f_2,f_3)+Y}. \end{aligned}$$

## Soliton-type solutions

Soliton-like solutions of Eq. (7) can be derived in the specific scenario where the modulus $\varrho$ approaches 1. This is exemplified as follows

73 $$\begin{aligned} \Psi _1^2(x,t)=\pm \frac{1}{\sqrt{2}}i+\eta _1\tanh (x-ct), \end{aligned}$$

74 $$\begin{aligned} \Psi _3^2(x,t)=\pm \frac{1}{\sqrt{2}}i+\eta _1\textrm{sech} \,(x-ct), \end{aligned}$$

75 $$\begin{aligned} \Psi _5^2(x,t)=\pm \frac{1}{\sqrt{2}}i+\eta _1\coth (x-ct), \end{aligned}$$

76 $$\begin{aligned} \Psi _{11}^2(x,t)=\pm \frac{1}{\sqrt{2}}i+\eta _1\textrm{csch} \,(x-ct), \end{aligned}$$

77 $$\begin{aligned} \Psi _{13}^2(x,t)=\pm \frac{1}{\sqrt{2}}i+\eta _1(\coth (x-ct)\pm \textrm{csch} \,(x-ct)), \end{aligned}$$

78 $$\begin{aligned} \Psi _{16}^2(x,t)=\pm \frac{1}{\sqrt{2}}i+\eta _1(\coth (x-ct)\pm i\,\textrm{sech} \,(x-ct)), \end{aligned}$$

79 $$\begin{aligned} \Psi _{19}^2(x,t)=\pm \frac{1}{\sqrt{2}}i+\eta _1(\tanh (x-ct)\pm i\,\textrm{sech} \,(x-ct)), \end{aligned}$$

80 $$\begin{aligned} \Psi _{23}^2(x,t)=\pm \frac{1}{\sqrt{2}}i+\eta _1(\textrm{sech} \,(x-ct)\pm \textrm{sech} \,(x-ct)), \end{aligned}$$

81 $$\begin{aligned} \Psi _{26}^2(x,t)=\pm \frac{1}{\sqrt{2}}i+\eta _1\sqrt{\frac{-Y}{2X}}\tanh \bigg (\sqrt{\frac{-Y}{2}}(x-ct)\bigg ), \end{aligned}$$

82 $$\begin{aligned} \Psi _{27}^2(x,t)=\pm \frac{1}{\sqrt{2}}i+\eta _1\sqrt{\frac{Y}{X}}\textrm{sech} \,\bigg (\sqrt{-Y}(x-ct)\bigg ), \end{aligned}$$

83 $$\begin{aligned} \Psi _{34}^2(x,t)=\pm \frac{1}{\sqrt{2}}i+\eta _1\frac{\textrm{sech} \,^2(x-ct)}{\tanh ^2(x-ct)+1}, \end{aligned}$$

84 $$\begin{aligned} \Psi _{36}^2(x,t)=\pm \frac{1}{\sqrt{2}}i+\eta _1\frac{\tanh (x-ct)}{1\pm \textrm{sech} \,(x-ct)}, \end{aligned}$$

85 $$\begin{aligned} \Psi _{40}^2(x,t)=\pm \frac{1}{\sqrt{2}}i+\eta _1\frac{\tanh (x-ct) \,\textrm{sech} \,(x-ct)}{\tanh ^2(x-ct)+(1+\varrho )\textrm{sech} \,(x-ct) -1-\varrho }, \end{aligned}$$

86 $$\begin{aligned} \Psi _{41}^2(x,t)=\pm \frac{1}{\sqrt{2}}i+\eta _1\frac{\tanh (x-ct) \,\textrm{sech} \,(x-ct)}{\tanh ^2(x-ct)+(-1+\varrho )\textrm{sech} \,(x-ct) -1-\varrho }, \end{aligned}$$

87 $$\begin{aligned} \Psi _{43}^2(x,t)=\pm \frac{1}{\sqrt{2}}i+\eta _1\frac{\sqrt{\frac{B^2+C^2-C^2}{B^2+C^2}}+\textrm{sech} \,(x-ct)}{B\,\tanh (x-ct)+C\,\,\textrm{sech} \,(x-ct)}. \end{aligned}$$

It is pertinent to note that the exact solutions $\Psi _{1}$, $\Psi _{2},~\cdots \Psi _{52}$ are derived and presented in Eqs. (21)–(72), where the choice of the positive (+ve) and negative (−ve) signs leads to distinct solutions. Additionally, it is worth highlighting that each exact solution provided in Eqs. (21)–(72) can be bifurcated into two solutions by selecting the positive and negative signs, although these variations have not been explicitly computed. Moreover, it should be emphasized that all the exact solutions outlined in Eqs. (21)–(72) can be validated through substitution. Notably, some of these solutions exhibit the incorporation of free parameters, namely *X*,  *Y*, and *Z*.

## Trigonometric-function solutions

Trigonometric-function solutions for Eq. (7) can be derived in the specific scenario where the modulus $\varrho$ approaches 0. For instance,

88 $$\begin{aligned} \Psi _{5}^2(x,t)=\pm \frac{1}{\sqrt{2}}i+\eta _1\csc (x-ct), \end{aligned}$$

89 $$\begin{aligned} \Psi _{6}^2(x,t)=\pm \frac{1}{\sqrt{2}}i+\eta _1\sec (x-ct), \end{aligned}$$

90 $$\begin{aligned} \Psi _{9}^2(x,t)=\pm \frac{1}{\sqrt{2}}i+\eta _1\tan (x-ct), \end{aligned}$$

91 $$\begin{aligned} \Psi _{11}^2(x,t)=\pm \frac{1}{\sqrt{2}}i+\eta _1\cot (x-ct), \end{aligned}$$

92 $$\begin{aligned} \Psi _{13}^2(x,t)=\pm \frac{1}{\sqrt{2}}i+\eta _1(\csc (x-ct)\pm \cot (x-ct)), \end{aligned}$$

93 $$\begin{aligned} \Psi _{14}^2(x,t)=\pm \frac{1}{\sqrt{2}}i+\eta _1(\sec (x-ct)\pm \tan (x-ct)), \end{aligned}$$

94 $$\begin{aligned} \Psi _{22}^2(x,t)=\pm \frac{1}{\sqrt{2}}i+\eta _1\frac{\sin (x-ct)}{2}, \end{aligned}$$

95 $$\begin{aligned} \Psi _{24}^2(x,t)=\pm \frac{1}{\sqrt{2}}i+\eta _1(\csc (x-ct)\pm \cot (x-ct)), \end{aligned}$$

96 $$\begin{aligned} \Psi _{32}^2(x,t)=\pm \frac{1}{\sqrt{2}}i+\eta _1\frac{(x-ct)\,\cos (x-ct)}{A(1+\sin (x-ct))}, \end{aligned}$$

97 $$\begin{aligned} \Psi _{36}^2(x,t)=\pm \frac{1}{\sqrt{2}}i+\eta _1\frac{\sin (x-ct)}{1\pm \cos (x-ct)}, \end{aligned}$$

98 $$\begin{aligned} \Psi _{37}^2(x,t)=\pm \frac{1}{\sqrt{2}}i+\eta _1\frac{\cos (x-ct)}{1\pm \sin (x-ct)}, \end{aligned}$$

99 $$\begin{aligned} \Psi _{42}^2(x,t)=\pm \frac{1}{\sqrt{2}}i+\eta _1\frac{\sqrt{\frac{B^2-C^2}{B^2}}+\sin (x-ct)}{B\,\cos (x-ct)+C}, \end{aligned}$$

100 $$\begin{aligned} \Psi _{43}^2(x,t)=\pm \frac{1}{\sqrt{2}}i+\eta _1\frac{\sqrt{\frac{B^2-C^2}{B^2}}+\cos (x-ct)}{B\,\sin (x-ct)+C}, \end{aligned}$$

101 $$\begin{aligned} \Psi _{44}^2(x,t)=\pm \frac{1}{\sqrt{2}}i+\eta _1\frac{\sqrt{\frac{B^2+C^2}{B^2+C^2}}+1}{B\,\sin (x-ct)+C\,\,\cos (x-ct)}. \end{aligned}$$

## Solitonic dynamics of the cdCH Eq. (7)

This section incorporates a graphical depiction of the attained results and corresponding physical explanations. The determination of exact solutions for the proposed model holds pivotal importance in elucidating diverse waveform manifestations within nonlinear complex structures. Utilizing the prescribed methodologies, the exact solutions are extracted and visually represented in multiple-soliton, soliton, trigonometric, hyperbolic, periodic, Jacobi’s elliptic, and singular wave functions. A *soliton*^23^, alternatively designated as a solitary wave, manifests as a self-sustaining wave packet that preserves its configuration during uniform propagation. The genesis of solitons is contingent upon the equilibrium of nonlinear and dispersive influences within the medium. These solitons serve as solutions to a broad category of weakly nonlinear dispersive partial differential equations integral to the modeling of physical and engineering systems. Conversely, a periodic traveling wave emerges as a periodic function in one dimension progressing at a consistent velocity, representing a distinctive spatiotemporal oscillation wherein both spatial and temporal dimensions exhibit periodic behavior. Diverse mathematical equations rely on periodic traveling waves, encompassing self-oscillatory, excitable, and reaction-diffusion-advection systems. Moreover, it is noteworthy that the parameter selection significantly influences the physical characteristics of the derived solutions. To provide a visual insight into these physical properties, 3D, and 2D graphs are generated. These graphical representations contribute to a comprehensive understanding of the observed phenomena.

**Figure 1:**
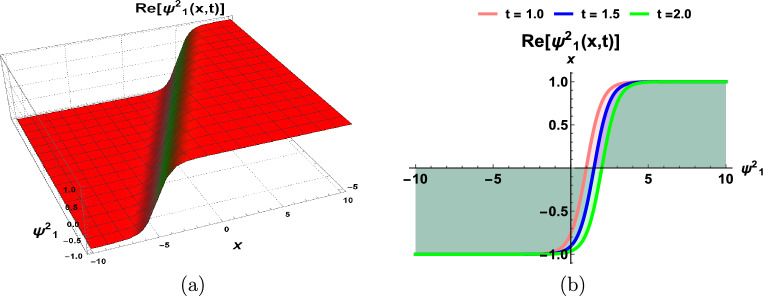
Dynamics of soliton-type solution $\Psi ^2_1(x,t)$ of cdCH Eq. (7) by using $\eta _1=1$ and $c=1$.

**Figure 2:**
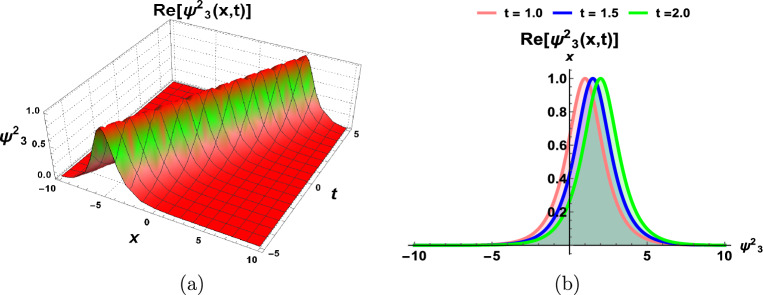
Dynamics of soliton-type solution $\Psi ^2_3(x,t)$ of cdCH Eq. (7) by the soliton-type solution $\Psi ^2_3(x,t)$ by using $\eta _1=1$ and $c=1$.

**Figure 3:**
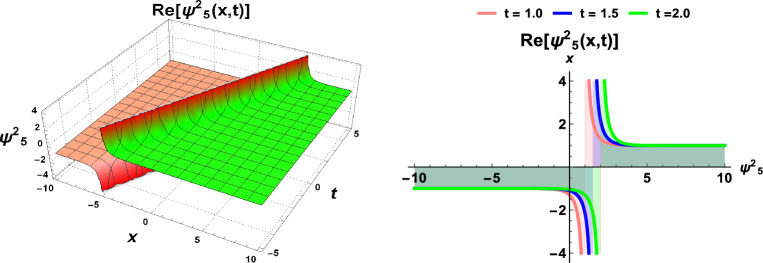
Dynamics of soliton-type solution $\Psi ^2_5(x,t)$ of cdCH Eq. (7) by using $\eta _1=1$ and $c=1$.

**Figure 4:**
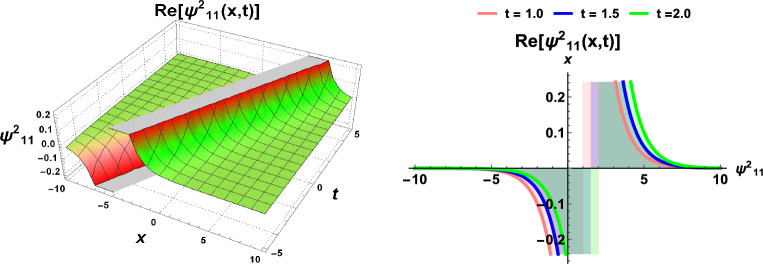
Dynamics of soliton-type solution $\Psi ^2_{11}(x,t)$ of cdCH Eq. (7) by using $\eta _1=1$ and $c=1$.

**Figure 5:**
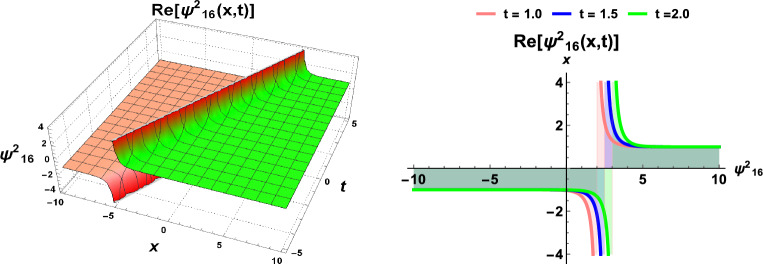
Dynamics of soliton-type solution $\Psi ^2_{16}(x,t)$ of cdCH Eq. (7) by using $\eta _1=1$ and $c=1$.

**Figure 6:**
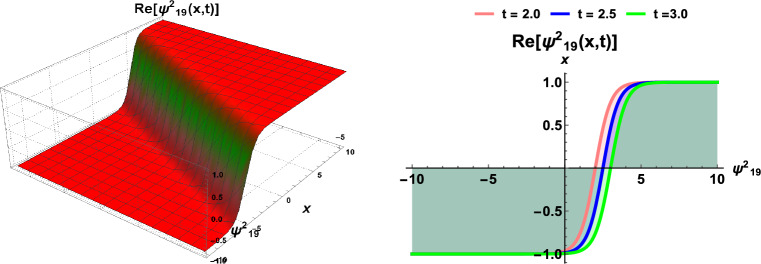
Dynamics of soliton-type solution $\Psi ^2_{19}(x,t)$ of cdCH Eq. (7) by using $\eta _1=1$ and $c=1$.

Figures 1 and  2, contingent upon the judicious selection of parameters, delineate the kink-type and bell-type soliton solutions, respectively. Figure 3 explicates the explicit representation of solitary waves, while Fig. 4 illustrates the singular soliton solution. Moreover, Fig. 5 presents the composite singular soliton solution, and Fig. 6 showcases the complex combo soliton solution. The outcomes of this endeavor are poised to serve as a fount of inspiration and motivation for forthcoming discussions spanning diverse research domains, particularly within the purview of solids engineering.

## Conclusions

The F-expansion method has been adeptly employed to derive fifty-two distinct exact solutions classified by the auxiliary equation $G'(\delta ) = XG^4(\delta ) +YG^2(\delta )+Z$ for the cdCH equation. This mathematical technique holds a notable advantage over alternative methods by encompassing all categories of exact solutions, encompassing Jacobi-elliptic and Weierstrass-elliptic functions. Moreover, it has yielded soliton-like solutions and trigonometric-function solutions as particular instances. The efficacy of the method in offering a diverse array of exact solutions, including those rooted in advanced mathematical functions, underscores its utility in addressing complex nonlinear PDEs, particularly in modeling phase separation dynamics in materials science.

The paper expanded its scope to encompass soliton-like and trigonometric-function solutions as special cases. This demonstrated that the outcomes previously achieved using the recently extended direct algebraic method (Rehman et al.)^24^, the modified auxiliary equation method (Lu et al.)^25^, the unified method (Adel et al.)^26^, and the modified simple equation method (Riaz et al.)^27^ were specific instances that fell within the more comprehensive context of the current findings. This method can also be further applied to certain NLEEs.

## Data Availability

All data generated or analyzed during this study are included in this published article.
